# What Survivors of Sexual Violence Want When Disclosing Their Experiences in Person or Online: Qualitative Interview Analysis

**DOI:** 10.2196/73497

**Published:** 2025-09-05

**Authors:** Elizabeth D Mayer, Roselyn Peterson, Ellie Kim, Prachi H Bhuptani, Reina Kiefer, Margarita Cruz-Sánchez, Lindsay Orchowski

**Affiliations:** 1 Department of Psychiatry & Behavioral Health Rhode Island Hospital Providence, RI United States; 2 Warren Alpert Medical School Brown University Providence, RI United States; 3 Department of Psychology University of Rhode Island Kingston United States

**Keywords:** #MeToo, sexual violence, online disclosure, social support, social reactions

## Abstract

**Background:**

Survivors of sexual victimization face a critical juncture when disclosing their experiences. How others react to their disclosure can significantly influence survivors’ psychological well-being.

**Objective:**

We aimed to address how survivors of sexual victimization would like to be supported when disclosing their experiences either in person or online.

**Methods:**

We conducted qualitative interviews with 51 participants who had experienced sexual victimization and disclosed their experiences either in person or online. Thematic analysis was applied to identify survivors’ perceptions of ideal and unhelpful responses to their disclosures in both in-person interactions and online environments.

**Results:**

When disclosing in person, survivors reported seeking acknowledgment, reassurance of support, and relief from self-blame. Survivors also preferred not to receive highly emotional reactions, unsolicited advice, or expressions of pity. Mutual disclosure was seen as validating and helpful by some, whereas others found it to be problematic, as it may diminish the courage it took for them to share their experiences. When disclosing online, survivors generally reported finding mutual disclosure helpful, as it fostered a sense of solidarity. Ideal responses to online disclosures included private messages of support and emotional support. However, judgment and probing for details were considered unsupportive reactions to online disclosure.

**Conclusions:**

Given that face-to-face interactions vary widely from the way in which we interact with others online, it follows that the way in which survivors seek to gain support from others varies across these 2 contexts. The findings of this study underscore the need for interventions aimed at educating individuals on how to provide support to survivors of sexual victimization, both in person and online.

## Introduction

### Background

Sexual violence is a prevalent issue in the United States and presents a major public health concern. Nearly 44% of women and 25% of men experience some form of unwanted sexual contact in their lifetime, with 21% to 29% of women and 3% of men experiencing attempted or completed rape in their lifetime [1]. Sexual victimization has profound and long-lasting effects on mental health, including strong associations with the development of posttraumatic stress disorder (PTSD), depression, anxiety, and problematic drinking behaviors [2-4]. Therefore, it is vital to understand how best to support survivors during the recovery process.

Reactions to the disclosures of sexual victimization significantly influence the psychological outcomes and overall well-being of survivors [5,6]. Responses to the disclosure of sexual victimization can be broadly categorized as either positive or negative, each bearing different implications for survivors’ recovery. Positive reactions, such as offering tangible aid or emotional support, have shown varying degrees of psychological benefits following in-person disclosures [7]. Although some studies have observed reductions in PTSD symptoms, others have found no impact of positive social reactions on psychological distress [8,9]. Conversely, negative reactions, such as blaming the survivor or attempts to dictate the survivor’s decisions, were found to be correlated with heightened psychological distress, as well as increased feelings of shame and rejection [10-12]. Negative social reactions can have a silencing effect, causing some survivors to feel reluctant to share their experiences with others in the future [13]. Furthermore, additional research by Ullman and Filipas [14] found that those who experienced assaults by strangers and sustained more injuries were more inclined to seek help from formal support services. However, they often encountered more negative responses from these health care providers [14]. In addition, while positive reactions were generally consistent across both formal and informal sources, formal health care providers were more likely to offer tangible aid, underscoring the importance of minimizing negative reactions within formal support systems to better assist survivors [14]. The seemingly straightforward categorization of reactions as either positive or negative fails to capture the complexity of how survivors experience and interpret such responses.

It is important to note that survivors’ perceptions of reactions to their sexual violence disclosure are deeply nuanced. Although survivors of sexual victimization generally agree as to what types of reactions to their disclosures are helpful, there are mixed findings on what characterizes harmful reactions [11]. For example, some reactions traditionally categorized as negative, including seeking revenge against the perpetrator, telling the survivor to move on, and attempting to control the survivor’s decisions, are perceived by some survivors as helpful and by others as hurtful [10]. Furthermore, the context in which a disclosure is given may influence the type of response a survivor finds most helpful in that moment. Previous research has found disclosure to be seen as more supportive when an encouraging context or climate in which disclosure can occur is fostered [6]. Furthermore, it is important to note that therapeutic contexts should create opportunities for disclosure by identifying the needs of the person disclosing [6]. These findings highlight the value of using qualitative methods to elucidate how survivors of sexual victimization describe the process of disclosure in their own words.

While research on online disclosures is growing, previous studies have primarily focused on in-person disclosures of sexual victimization. The rise of social media and movements such as #MeToo have broadened the scope, prompting further exploration into how survivors share their experiences in digital spaces. Initially started by the activist Tarana Burke in 2006, the #MeToo movement gained momentum when, in response to Harvey Weinstein’s sexual assault allegations, Alyssa Milano asked others via a tweet to share their experiences online [15]. Her call to action gained massive attention across the globe, highlighting the widespread issue of sexual harassment and assault [16]. Because of the increasing prevalence of social media as platforms for disclosure, there are now a growing number of studies that seek to understand the context and repercussions of online reactions to disclosure of sexual victimization.

The impact of online disclosure has revealed a complex landscape of both positive and negative outcomes for survivors. For instance, many survivors report experiencing a sense of empowerment through online disclosure, citing benefits such as reduced social isolation, increased community support, and the ability to reclaim their narrative [17-19]. However, online disclosure also presents significant challenges. For example, survivors frequently encounter digital harassment, victim blaming, and hostile reactions that can further impact trauma symptoms [20-22]. Considering the rise of online disclosure of sexual violence across social media platforms and the large-scale impact of #MeToo, additional research that explores the nuanced disclosure experiences of those involved in the movement is critical for understanding the evolving landscape of online disclosure.

Sexual victimization is often not disclosed or reported, with many survivors facing significant barriers to sharing their experiences with both formal and informal support systems [23,24]. These barriers may include fear of judgment, concerns about not being believed, feelings of shame, and anticipated negative reactions [25,26]. Although the #MeToo movement empowered many individuals to speak out, witnessing the backlash or harmful responses to others’ disclosures can discourage survivors from coming forward [13,27,28]. Importantly, even those who choose not to disclose still offer valuable perspectives on the types of responses they would hope to receive if they were to share their experiences either online or in person.

### Present Study

In sum, the way individuals respond to disclosures of sexual victimization, whether in person or online, is crucial in shaping survivors’ psychological outcomes [29]. Understanding the characteristics of effective and supportive social reactions from the perspective of survivors is important for developing interventions to promote healing after sexual violence. Existing research has shown that reactions to disclosures greatly influence posttraumatic adjustment and has explored qualitative perspectives to understand helpful and harmful reactions [11,29,30]. In light of the increase in the number of studies seeking to better understand the context and consequences of sharing sexual victimization experiences online [25,31], this study moves the field forward by engaging with survivors directly to understand ideal reactions to online disclosure via #MeToo. Specifically, the primary research question asked how survivors described ideal responses to disclosures of sexual violence in both in-person and online contexts. Accordingly, we applied thematic content analysis to survivors’ perceptions of ideal reactions to actual or hypothetical disclosures of sexual victimization, either in person or online, via the #MeToo movement.

## Methods

### Participants

Participants were recruited for a study on the disclosure of unwanted sexual experiences via social media platforms. Participants were invited from a larger quantitative study examining the impact and disclosure of sexual violence within the context of the #MeToo movement. The inclusion criteria required participants to be aged >18 years and to have experienced sexual violence from the age of 14 years to the time of the study. Participants completed the Sexual Experiences Survey–Short Form Victimization to confirm eligibility [32,33]. At the conclusion of the larger survey, participants were asked if they would like to take part in a qualitative interview. Out of the total sample (N=767), 6.6% (n=51) agreed to be interviewed about their disclosures of sexual violence. From the interviews with the 51 participants, 27.5% (n=14) had not disclosed their experience of sexual violence, 39.2% (n=20) had reported only disclosing it in person, and 33.3% (n=17) had disclosed their experiences of sexual violence both in person and online via the #MeToo movement, forming the current analytic sample. Participants were asked what their ideal disclosure responses were to both their lived disclosures and hypothetical disclosures if they had not disclosed online or had not disclosed in any context.

### Procedures

Data collection was conducted from February 2020 to February 2022, with detailed procedures described in previous studies (Driessen et al [34] and Peterson et al [35]). Participants were recruited throughout the United States via social media advertisements on platforms such as Facebook, Instagram, and X (formerly known as Twitter). Participants provided consent before accessing the online survey materials and received a US $10 Amazon gift card upon completing the survey as compensation. Participants completed a self-reported survey assessing the characteristics of their sexual violence disclosure or disclosures, the type of reaction they received, along with measures related to posttraumatic stress symptoms and substance use. At the end of the survey, participants indicated their interest in participating in an interview, after which the research team contacted them. The individual interviews lasted 30 minutes and were conducted virtually by 1 of 3 trained female interviewers. Interviews were audio recorded and transcribed verbatim. Demographic characteristics were collected at the start of the interviews, which followed a semistructured guide designed to examine online and in-person disclosure of sexual victimization, the reactions received upon disclosure, and participants’ perceptions of reactions. Responses to the following interview questions were considered for the present analysis: (1) how do you wish people had reacted to you when you shared your experience, (2) how do you wish people would react if you shared your experience, (3) how do you wish people had reacted online when you shared your experience, and (4) how do you wish people would react if you shared your experience online? Participants were asked only these questions that were relevant to their reported disclosure experience. For example, participants who disclosed their experiences in person but not online were asked questions 1 and 4. The interviews were conducted until the research team concluded that theoretical saturation had been reached. At this point, no new concepts or themes emerged from the data, and additional data collection no longer provided novel codes [36-38]. Although the interview agenda asked about ideal reactions to sexual violence disclosure, participants described both desired and undesired responses. Themes reflecting both desired and undesired responses were incorporated into the initial development of the codebook.

All interviews were conducted by female research staff with master- and doctoral-level training in psychology and experience working with survivors of sexual violence. The coding team consisted of women with diverse professional backgrounds and shared expertise in violence prevention and intervention research. Team members included both junior and senior research faculty with doctoral training in clinical psychology, as well as research staff with bachelor-level training in psychology. The team’s collective background informed both the analytic process and interpretation of the findings, while ongoing discussions during coding helped to surface and reflect on potential biases and assumptions.

### Ethical Considerations

All study procedures were approved by the Rhode Island Hospital institutional review board (1472511). Before beginning the online survey, the participants reviewed an informed consent page and were prompted to indicate if they consented to the study. To protect anonymity, the study received a waiver of written consent documentation. All data were collected anonymously, and no names or contact information were linked to participants’ responses. Participants received US $10 for completing the initial quantitative survey and US $30 for the qualitative interview. All compensation was provided in the form of Amazon gift cards.

### Data Analysis Plan

Data analysis was conducted using applied thematic analysis [39] by a team of 5 analysts (EDM, RP, RK, MSC and PHB). The coding structure was developed based on the interview agenda (deductive) and topics raised by the participants (inductive). The coding team began analyzing transcripts as a group to establish a firm shared understanding of the codebook and to ensure that there were no further edits to the codebook. Once a strong shared understanding was achieved and the codebook was finalized, the team transitioned to paired coding. Two coders independently analyzed each transcript and resolved discrepancies through discussion, ultimately achieving 100% agreement within the coding process. Finalized codes were entered into NVivo (Lumivero) qualitative data software [40]. The coding team met regularly to discuss codes, clarify definitions, and monitor use to enhance data trustworthiness. The first and second authors reviewed, summarized, and interpreted the codes relevant to the study’s research questions through multiple readings of both the codes and raw data. Discrepancies were addressed collaboratively by engaging in discussion, posing clarifying questions, and critically examining the most illustrative quotes for each theme. The team ultimately reached a shared consensus on the codes and supporting data. Representative quotes were selected to exemplify the findings presented in the subsequent sections.

## Results

### Overview

Of the 51 participants, the average age of the sample was 29 (range 19-59) years. Most participants (n=42, 82%) identified as cisgender women, just under half (n=23, 43%) identified as heterosexual, and the majority (n=38, 70%) identified as White. Table 1 provides a detailed demographic breakdown of the sample. Of the 37 participants who disclosed their experience of sexual violence, the most common disclosure recipients were friends (n=30, 81%), family (n=25, 68%), partners (n=25, 68%), and medical and mental health providers (n=15, 41%). Other disclosure recipients were categorized as acquaintances (n=8, 22%), law enforcement (n=5, 14%), and educators (n=5, 14%).

**Table 1:** Demographic characteristics of the study sample (N=51).

Sample characteristics		Participants, n (%)
**Gender**		
	Cisgender woman	42 (82)
	Nonbinary	3 (6)
	Transgender man	2 (4)
	Genderqueer or bigender	2 (4)
	Cisgender man	1 (2)
	Two spirit	1 (2)
**Sexual orientation**		
	Heterosexual	23 (45)
	Bisexual	10 (20)
	Queer	5 (10)
	Pansexual	4 (8)
	Gay	1 (2)
	Lesbian	1 (2)
	Demisexual	1 (2)
	Questioning	1 (2)
	Not heterosexual	1 (2)
	Prefer not to answer	4 (8)
**Ethnicity**		
	American Indian	3 (6)
	Black or African American	1 (2)
	Indian	2 (4)
	Latino or Hispanic	1 (2)
	Multiracial	3 (6)
	White	38 (75)
	Prefer not to answer	3 (6)

The findings are organized according to the coding structure, beginning with supportive and unsupportive reactions and mutual disclosure in response to in-person disclosure, followed by the same categories in response to online disclosure. Illustrative quotes were selected based on their ability to represent key themes. When disclosing in person, survivors seek *acknowledgment, reassurance of support,* and *relief from self-blame* and do not want to receive *highly emotional reactions*, *unrequested guidance,* or *expressions of pity*. For those who disclosed *in person*, mutual disclosure was seen as *validating* and *helpful* by some, while others found it to be *problematic,* as it may diminish the courage it took for them to share their experiences. Perceived ideal responses to online disclosures include *private messages of support* in addition to *emotional support*. Survivors who disclosed online found that *mutual disclosure* helped foster a *sense of solidarity*. In contrast, online reactions, such as expressions of judgment and probing for details, were regarded by survivors as unsupportive.

### Supportive Reactions to In-Person Disclosure

When prompted about ideal reactions when disclosing in person, participants expressed the importance of acknowledging both the unwanted experience itself and the hardship they have since endured. One participant noted that acknowledgment helps them to feel empowered as a survivor:

Getting that acknowledgement of you’ve gone through it, you survived it and now what you’re able to do to help other people shows that you’re not broken.

Cisgender woman aged 21 y; disclosed in person only

Survivors expressed the desire to be reassured that they are supported regardless of how they choose to address the assault moving forward (ie, reporting or seeking formal support). Here, survivors expressed that they appreciate having the space to communicate how they would like to be supported as opposed to the disclosure target assuming how best the survivor could be supported:

Reassurance that like whoever you’re telling is there for you, and there to support you in whatever action you decide to take, whether that be to not tell anyone else or to move forward with pressing charges or whatever action that the person wants to take, I think just knowing that you’re being supported no matter what you do.

Cisgender woman aged 23 y; did not disclose

It can be tempting for disclosure targets to express what they think the survivor should do to address the situation. However, interviews revealed that unless explicitly stated that they want help reporting or accessing other resources, they are more so hoping to be heard and validated during disclosure interactions. Overwhelmingly, survivors stated that they wanted to hear the words “It’s not your fault.” Survivors noted that self-blame and shame often arise after experiencing sexual violence. When sharing experiences with others, hearing the disclosure target say that they are not responsible for the harm they experienced can help to move past unhelpful thoughts of self-blame:

I think hearing a lot of times that it wasn’t my fault and that I didn’t deserve it. And it’s a horrible thing that never should have happened in the first place and that people wish that they could have done something to prevent it helps.

Cisgender woman aged 21 y; disclosed in person only

This quote highlights that by affirming that the survivor is not at fault and further and they did not deserve to experience harm, it can help navigate the challenging feelings that can arise in the wake of sexual violence.

### Unsupportive Reactions to In-Person Disclosure

The 3 most prominent unwelcome reactions to in-person disclosure were unrequested opinions or advice, highly emotional reactions, and expressions of pity. Participants expressed that receiving questions that probed for details contributed to a sense of invalidation. For example, one participant described the impact of receiving unwelcome opinions:

There have been times when people have been like, “no, like what you experienced, it was this” ...one friend was like, “well, technically what you experienced was like rape” And I’m like, oh no. I mean, I just wasn’t ready to accept that yet.

Cisgender woman aged 26 y; disclosed in person only

Many survivors also reported that overly emotional reactions, such as extreme sadness, anger, or sobbing, take the focus away from the initial disclosure and ultimately make the survivor feel worse. Unsupportive reactions also put the survivors in a position where they feel the need to console the disclosure target when they were the ones looking for support in the first place:

I think the most helpful reactions have been sort of the most matter of fact ones...I feel like when people break down crying or are just very emotional about it, it’s made me just feel like I have caused that pain.

Cisgender woman aged 26 y; disclosed in person only

Furthermore, many participants noted that expressions of pity made them feel worse about themselves as opposed to empowered:

One thing I really wish was more of a norm is that I wish people wouldn’t immediately feel like they have to tell me something or pity me.

Cisgender woman aged 22 y; disclosed in person only

The desire to immediately console or express sorrow for what the survivor experienced may be well intentioned but, as expressed by this participant, can ultimately be an unhelpful reaction. Survivors often emphasized the importance of being met with calm, composed, and empathetic responses that prioritize their needs over the disclosure target’s emotional reaction. While expressions of care and concern are valuable, maintaining a focus on the survivor’s experience and providing steady support helps create a safe space for them to process their emotions without feeling burdened by the need to manage someone else’s feelings.

### Mutual Disclosure in the Context of In-Person Disclosure

There were mixed opinions on mutual disclosure as a response to in-person disclosures. Some participants expressed that if a disclosure target had a similar experience, it would be helpful to know such information to foster a deeper level of understanding and reduce feelings of isolation:

I feel like the experiences that some people had shared in return were also nice in the sense that you know, you know that other people who share understand what you’re going through so like you’re not alone. It still feels like very lonely path to walk.

Transgender man aged 25 y; disclosed in person only

Fewer survivors felt that mutual disclosure was unhelpful; however, participants highlighted important nuances of both in-person and online disclosure responses. One survivor expressed that it takes an immense amount of courage to disclose to someone and that mutual disclosure can take away from the importance of the moment:

I think that a lot of people...feel compelled to also disclose back if they’ve had some sort of experience. I think that can be kind of problematic because it might have taken a lot for this person to like say and articulate what happened and like to have their moment taken away from them by mutual disclosure is...I think it could be problematic.

Cisgender woman; disclosed in person

Thus, whereas mutual disclosure can offer essential validation and a sense of solidarity among survivors, some may feel it diminishes the personal significance of an individual’s disclosure.

### Supportive Reactions to Online Disclosure

Participants who disclosed online as a part of the #MeToo movement articulated that, despite posting their experience publicly, receiving private messages of support helped to establish a sense of acknowledgment; one participant noted the following:

I think I would have just liked personal DMs of people telling me that it wasn’t my fault and how strong I was and people just telling me that they had my back and they were on my side.

Cisgender woman aged 21 y; disclosed in person

One participant noted they wished people acknowledged their disclosure directly after seeing it on social media:

I think that would have been helpful if I got more messages...more acknowledging it or if like people ignored it on social media then they reached out to me in other ways...and did more of a private acknowledgment. I think more acknowledgment of it would have been beneficial to me.

Cisgender woman aged 23 y; disclosed in person and online

Personal messages helped to build a sense of acknowledgment and validation for the survivor. While posting publicly can be an empowering act, participants expressed that private messages of support added a crucial layer of emotional connection and validation that public comments often lack. These messages reassured survivors that their experiences were seen, believed, and supported, fostering a sense of solidarity and reducing feelings of isolation.

### Unsupportive Reactions to Online Disclosure

Participants highlighted that receiving judgmental comments or questioning their experience are considered unwelcome reactions when disclosing online. Survivors emphasized that it is inappropriate for anyone to question the experiences that have been shared online:

I feel like at the end of the day no one should be questioning anyone’s experience, and it’s not, especially if like, someone’s sharing their story, it’s not up to like, a person, or like, there shouldn’t be an environment created where we continuously question what specifically happened.

Genderqueer participant aged 19 y; did not disclose

This quote underscores how questioning or disbelief can create an unsupportive environment that undermines the core elements of an empathetic response.

In addition, participants conveyed that reactions of judgment or disbelief were considered unsupportive responses. One participant specifically stated that they find online reactions to be uncomforting and that most comments tend to come across as judgmental:

I’ve never found reactions online to be comforting...people just commenting what they think on a post that’s so personal, it gets a bit more judgmental or the possibility to be more judgmental.

Cisgender woman aged 29 y; disclosed in person and online

The lack of control over who engages with their posts, coupled with the anonymity and permanence of online platforms, makes survivors particularly vulnerable to unsupportive reactions. This further reinforces the importance of creating a supportive and respectful online environment, where survivors can share their experiences without fear of judgment or invalidation.

### Mutual Disclosure in the Context of Online Disclosure

In the context of online disclosures, receiving a mutual disclosure was mentioned solely as a welcomed reaction. Participants expressed that, although they hope others do not have to endure similar experiences, mutual disclosure provides reassurance that they are not alone. In the context of online disclosure, one participant expressed the following:

I think if someone were to also say this happened to me too...it makes me feel...like, I’m not alone...it’s just good to know that I’m in the company of people who struggle with the same thing.

Cisgender woman aged 40 y; disclosed in person

In addition, it was expressed that even when sharing an experience online, survivors felt that private messages of mutual disclosure are validating:

I feel like it would’ve been helpful if people private messaged me...because it can be helpful when I see other people who I see to be independent, strong people who have also been victimized. Obviously, I wish it didn’t happen to them, but like, “Oh, okay, this isn’t a me problem. This isn’t like a shortcoming of mine. This is like the other person’s problem.”

Cisgender woman aged 26 y; disclosed in person

When discussing hypothetical online disclosure reactions, the participant mentioned earlier expressed a sentiment of drawing strength from the survivor community, aligning with the experiences shared by individuals who disclosed their stories through the #MeToo movement. This highlights the empowering impact of connecting with other resilient individuals who have also been affected by sexual violence and how the online sphere lends itself to such connections.

## Discussion

### Principal Findings

This study aimed to qualitatively analyze survivors’ perceptions of supportive and unsupportive reactions to actual or hypothetical disclosures when disclosing sexual victimization, either in person or online, via the #MeToo movement. Data were collected from a relatively large qualitative sample of 51 participants who disclosed their experiences of sexual violence both in person and online via the #MeToo movement. The findings shed light on critical nuances in how survivors of sexual victimization perceive reactions to their disclosures, while also extending the existing literature by examining such themes within the context of the online realm.

There were mixed opinions surrounding the perception of mutual disclosure as a response to sharing an experience of sexual violence. In regard to online disclosures, ideal responses include private messages that offer emotional support, and many survivors reported mutual disclosure fostered a sense of solidarity, a finding echoed in the study by Driessen et al [34]. In the online #MeToo context, mutual disclosure was often highlighted as a positive response. However, while some consider mutual disclosure during in-person conversations to be validating and supportive, others perceive it to be problematic, feeling that it undermines the courage required to share their experiences. In a dyadic study examining disclosure dynamics between survivors and their support persons [41], it was found that pulling from traumatic experiences to respond to the survivor was considered a highly supportive reaction by survivors. Although support recipients did not always explicitly disclose their own experiences of sexual violence to the survivor, it was often the case that their responses were informed by personal histories of trauma [41]. In many cases, support providers chose not to share their trauma directly, instead drawing on their understanding of such experiences to offer compassionate and validating responses [41]. Studies, such as those by Gorissen et al [42] and Gueta et al [17], provide valuable insight into these motivations for disclosing sexual assault online, which often center on a desire to connect with others who can understand and may help to contextualize and either support or complicate the idea that lived experience is central to offering effective support. Thus, it should be noted that having lived experience is not a requirement for an empathic response to disclosure. However, this study did not examine disclosure motives; one key component behind the findings related to the positive perception of online mutual disclosure could be that the motive to share online is manifested in the hope of connecting with others who have had similar experiences.

The findings identified supportive reactions to in-person disclosures, including acknowledgment and reassurance of support and relief from self-blame. Unsurprisingly, survivors emphasized a desire for emotionally supportive disclosure responses. Participants wanted to avoid highly emotional reactions, unrequested guidance, or expressions of pity. The findings at hand are in line with existing research that explored helpful and unhelpful reactions to disclosure based on the relationship with the recipient and found that among disclosures to informal support providers, survivors desired supportive listening and antiblaming messaging [43]. Furthermore, the themes in our analysis are echoed in existing frameworks such as the Social Reactions Questionnaire [5], which provides a general framework for understanding positive reactions (emotional support and tangible aid) and negative reactions (controlling the survivor’s decisions, blaming, treating you differently, distraction, and egocentric behavior) to sexual violence disclosure.

Participants also highlighted simple statements, such as “it’s not your fault,” as helpful in reducing the burden of self-blame often associated with sexual violence regarding in-person disclosure. The same sentiment was echoed on the internet based space, as participants desired direct messages of support in response to their online #MeToo disclosures. Feelings of shame and self-blame are common in the wake of sexual violence and can be detrimental to the healing process [44,45]. Existing research has reinforced the importance of positive social reactions in reducing feelings of self-blame, PTSD symptomatology, and drinking to cope [9,29,46]. Furthermore, research conducted on cognitive processing therapy (CPT) for sexual victimization shows mechanisms of CPT (eg, modules on helping survivors restructure thoughts to reduce shame or guilt and recognize what they experienced was not their fault) have shown to be key components of treatment after sexual victimization [47]. The treatment mechanisms at work in CPT (eg, cognitive restructuring) are beneficial to survivors in that survivors are often alleviated of feelings, such as blame, guilt, and shame. By reassigning responsibility for what occurred onto the perpetrator, feelings that are more in line with recovery are instilled in the survivor (eg, empowerment and autonomy) [47]. The importance of dispelling shame is echoed in the findings from this study in that participants emphasized the importance of hearing from others that what they experienced was not their fault as being important after disclosure. However, it can be challenging to know exactly how people will respond to online disclosure, and oftentimes, it is not always positive.

Themes surrounding the negative online reactions highlighted judgment or intrusive questioning as detrimental responses to sexual violence disclosure. A major facet, and drawback, of disclosing in an online space is the sheer reach that posts can receive as they gain popularity and online traffic increases. This highlights the duality of online disclosures: while public posts can amplify awareness and solidarity on a broad scale, private, individualized responses are essential for providing survivors with the personal reassurance and acknowledgment they need to feel truly supported. The findings align with those of the study by Gundersen and Zaleski [19], who identified that positive reactions to online disclosure can be empowering, while also highlighting that some may have negative experiences, such as the detrimental effects of public judgment and intrusive questioning prevalent in digital spaces. Unfortunately, online disclosures open up to online trolling, which is the act of posting or commenting online with the intention of deliberately upsetting or disturbing others. Research surrounding the perception of trolling in response to #MeToo disclosure argues that trolling is understood as a natural consequence of online disclosures, stemming from power struggles due to clashing personal and political beliefs [48]. Participants in this study acknowledged that while they hope that online disclosures allow them to connect with others who can relate, it can be damaging to receive comments and messages from social media users who are questioning their experiences or spreading hateful messages. These findings highlight the pressing need for platform-specific strategies that reduce harm and foster supportive online spaces, particularly as survivors navigate the evolving landscape of digital disclosure and the shifting dynamics of social media.

### Limitations

This study had several limitations, the first of which being that we did not study the impact of the various disclosure responses, the relationship to the disclosure target, or how many times they had disclosed. We recognize that sexual violence disclosure is deeply nuanced, and many factors may influence what is perceived as an ideal disclosure. Furthermore, future studies may explore disclosure motives in tandem with perceived reactions to obtain a multifaceted understanding of what survivors desire in response to a disclosure. In addition, the study did not distinguish between the findings based on actual and hypothetical disclosure. It should be noted that survivors who have not yet disclosed their experiences still hold valuable perspectives on what constitutes supportive responses to sexual violence disclosures.

This study focused only on the #MeToo movement. While #MeToo had many positive impacts, most notably expanding awareness of the prevalence of sexual violence, it receives criticism for lacking an intersectional approach and leaving out diverse voices [49]. There are many other movements surrounding gender-based violence that capture specific cultural and social norms, such as #NiUnaMenos in Latin America, #SeAcabo in Spain, and most recently, the protests that have erupted in France following the events of Gisèle Pélicot’s public court case [50-52]. Culturally grounded movements provide important information on the nuanced experiences and challenges faced by different communities and highlight the importance of context in understanding how disclosures are received and the responses they elicit. By focusing solely on the #MeToo movement, this study may overlook critical insights from other important initiatives that address the complexities of gender-based violence across diverse populations. Future research should aim to incorporate a broader range of movements to provide a more comprehensive understanding of disclosure dynamics and to ensure that all voices are represented in the conversation about sexual violence.

### Conclusions

This study is one of the first to qualitatively explore the complexities of online reactions to sexual violence disclosures, addressing a critical gap in the existing literature on how online responses shape the experiences of survivors. By deepening our understanding of the nuances within the online environment, the findings can inform the development of targeted resources and interventions specifically designed for digital platforms. Such resources could include tools to connect survivors with trauma-informed support or provide guidelines for offering empathetic, respectful responses to sexual violence disclosures. Moreover, the insights gained from the study can contribute to the creation of positive, supportive online spaces that foster healing and resilience for survivors of sexual violence, ultimately promoting a more compassionate and informed digital landscape for disclosure and recovery.

## Abbreviations

CPT: cognitive processing therapy
PTSD: posttraumatic stress disorder

## Data Availability

Data from this study are not publicly available but are available from the corresponding author upon request.

## References

[1] Smith SG, Zhang X, Basile KC, Merrick MT, Wang J, Kresnow M, Chen J (2018). The national intimate partner and sexual violence survey : 2015 data brief – updated release. National Center for Injury Prevention and Control, Centers for Disease Control and Prevention.

[2] Blayney JA, Hequembourg A, Livingston JA (2021). Rape acknowledgment and sexual minority women's mental health and drinking behaviors. J Interpers Violence.

[3] Dworkin ER, Menon SV, Bystrynski J, Allen NE (2017). Sexual assault victimization and psychopathology: a review and meta-analysis. Clin Psychol Rev.

[4] Neilson EC, Bird ER, Metzger IW, George WH, Norris J, Gilmore AK (2018). Understanding sexual assault risk perception in college: associations among sexual assault history, drinking to cope, and alcohol use. Addict Behav.

[5] Ullman SE (2000). Psychometric characteristics of the social reactions questionnaire: a measure of reactions to sexual assault victims. Psychol Women Q.

[6] Ullman SE, Foynes MM, Tang SS (2010). Benefits and barriers to disclosing sexual trauma: a contextual approach. J Trauma Dissociation.

[7] Ahrens CE, Cabral G, Abeling S (2009). Healing or hurtful: sexual assault survivors' interpretations of social reactions from support providers. Psychol Women Q.

[8] Orchowski LM, Gidycz CA (2015). Psychological consequences associated with positive and negative responses to disclosure of sexual assault among college women: a prospective study. Violence Against Women.

[9] Ullman SE, Peter-Hagene L (2014). Social reactions to sexual assault disclosure, coping, perceived control and PTSD symptoms in sexual assault victims. J Community Psychol.

[10] Campbell R, Ahrens CE, Sefl T, Wasco SM, Barnes HE (2001). Social reactions to rape victims: healing and hurtful effects on psychological and physical health outcomes. Violence Vict.

[11] Dworkin ER, Brill CD, Ullman SE (2019). Social reactions to disclosure of interpersonal violence and psychopathology: a systematic review and meta-analysis. Clin Psychol Rev.

[12] Relyea M, Ullman SE (2015). Unsupported or turned against: understanding how two types of negative social reactions to sexual assault relate to post-assault outcomes. Psychol Women Q.

[13] Ahrens CE (2006). Being silenced: the impact of negative social reactions on the disclosure of rape. Am J Community Psychol.

[14] Ullman SE, Filipas HH (2001). Correlates of formal and informal support seeking in sexual assault victims. J Interpers Violence.

[15] Jaffe AE, Cero I, DiLillo D (2021). The #MeToo movement and perceptions of sexual assault: college students' recognition of sexual assault experiences over time. Psychol Violence.

[16] Sayej N (2017). Alyssa Milano on the #MeToo movement: 'we're not going to stand for it any more'. The Guardian.

[17] Gueta K, Klar-Chalamish C, Ullman SE (2024). The process of online disclosures of interpersonal victimization: a systematic review. Trauma Violence Abuse.

[18] Maier SL (2023). Rape victim advocates' perceptions of the #MeToo movement: opportunities, challenges, and sustainability. J Interpers Violence.

[19] Gundersen KK, Zaleski KL (2020). Posting the story of your sexual assault online: a phenomenological study of the aftermath. Feminist Media Stud.

[20] Gueta K, Eytan S, Yakimov P (2020). Between healing and revictimization: the experience of public self-disclosure of sexual assault and its perceived effect on recovery. Psychol Viol.

[21] Bogen KW, Orchowski LM, Ullman SE (2024). Online disclosure of sexual victimization and social reactions: what do we know?. Resistance & Recovery in the #MeToo era, Volume I.

[22] Stubbs-Richardson M, Rader NE, Cosby AG (2018). Tweeting rape culture: examining portrayals of victim blaming in discussions of sexual assault cases on Twitter. Fem Psychol.

[23] Carretta CM, Burgess AW, DeMarco R (2015). To tell or not to tell. Violence Against Women.

[24] Griffin VW, Wentz E, Meinert E (2022). Explaining the why in #WhyIDidntReport: an examination of common barriers to formal disclosure of sexual assault in college students. J Interpers Violence.

[25] Alaggia R, Wang S (2020). "I never told anyone until the #metoo movement": what can we learn from sexual abuse and sexual assault disclosures made through social media?. Child Abuse Negl.

[26] Ullman SE, O'Callaghan E, Shepp V, Harris C (2020). Reasons for and experiences of sexual assault nondisclosure in a diverse community sample. J Fam Violence.

[27] Bogen KW, Bleiweiss KK, Leach NR, Orchowski LM (2021). #MeToo: disclosure and response to sexual victimization on twitter. J Interpers Violence.

[28] Orchowski LM, Grocott L, Bogen KW, Ilegbusi A, Amstadter AB, Nugent NR (2022). Barriers to reporting sexual violence: a qualitative analysis of #WhyIDidntReport. Violence Against Women.

[29] Ullman SE (2023). Talking about sexual assault: society’s response to survivors, second edition. American Psychological Association.

[30] Edwards KM, Dardis CM, Sylaska KM, Gidycz CA (2015). Informal social reactions to college women's disclosure of intimate partner violence: associations with psychological and relational variables. J Interpers Violence.

[31] Jaffe AE, DiLillo D, Hoffman L, Haikalis M, Dykstra RE (2015). Does it hurt to ask? A meta-analysis of participant reactions to trauma research. Clin Psychol Rev.

[32] Johnson SM, Murphy MJ, Gidycz CA (2017). Reliability and validity of the sexual experiences survey–short forms victimization and perpetration. Violence Vict.

[33] Koss MP, Gidycz CA (1985). Sexual experiences survey: reliability and validity. J Consult Clin Psychol.

[34] Driessen MC, Bhuptani PH, Kiefer R, Peterson R, Mayer E, Cruz-Sanchez M, Weiss NH, Orchowski LM (2024). Reactions to and impact of survivor online disclosures: a qualitative analysis. J Child Sex Abus.

[35] Peterson R, Mayer E, Bhuptani PH, Kiefer R, Cruz‐Sánchez M, Kim E, Orchowski LM (2025). A qualitative analysis of the #MeToo online sphere. J Community Appl Soc Psychol.

[36] Guest G, Bunce A, Johnson L (2006). How many interviews are enough?: an experiment with data saturation and variability. Field Methods.

[37] Hennink MM, Kaiser BN, Marconi VC (2017). Code saturation versus meaning saturation: how many interviews are enough?. Qual Health Res.

[38] Saunders B, Sim J, Kingstone T, Baker S, Waterfield J, Bartlam B, Burroughs H, Jinks C (2018). Saturation in qualitative research: exploring its conceptualization and operationalization. Qual Quant.

[39] Guest G, MacQueen KM, Namey EE (2012). Applied Thematic Analysis.

[40] (2008). NVivo 8. QSR International.

[41] Lorenz K, Ullman SE, Kirkner A, Mandala R, Vasquez AL, Sigurvinsdottir R (2018). Social reactions to sexual assault disclosure: a qualitative study of informal support dyads. Violence Against Women.

[42] Gorissen M, van den Berg CJ, Bijleveld CC, Ruiter S, Berenblum T (2023). Online disclosure of sexual victimization: a systematic review. Trauma Violence Abuse.

[43] Kirkner A, Lorenz K, Ullman SE (2021). Recommendations for responding to survivors of sexual assault: a qualitative study of survivors and support providers. J Interpers Violence.

[44] Bhuptani PH, Messman-Moore TL, O’Donohue WT, Schewe PA (2019). Blame and shame in sexual assault. Handbook of Sexual Assault and Sexual Assault Prevention.

[45] Robinson MD, Hassija CM, Wellman JD (2024). Trauma-related shame, characterological self-blame, and psychological outcomes among sexual assault survivors. J Loss Trauma.

[46] Neilson EC, Gilmore AK, Pinsky HT, Shepard ME, Lewis MA, George WH (2015). The use of drinking and sexual assault protective behavioral strategies: associations with sexual victimization and revictimization among college women. J Interpers Violence.

[47] Resick PA, Galovski TE, Uhlmansiek MO, Scher CD, Clum GA, Young-Xu Y (2008). A randomized clinical trial to dismantle components of cognitive processing therapy for posttraumatic stress disorder in female victims of interpersonal violence. J Consult Clin Psychol.

[48] McWan B (2022). Trolling of# MeToo: audiences' perceptions. Bowling Green State University.

[49] Onwuachi-Willig A (2018). What about #UsToo?: the invisibility of race in the #MeToo movement. Yale Law J.

[50] Bedrosian A (2019). Write, tweet, march: how Argentina’s NiUnaMenos employed discourse in digital and physical spaces to reach the masses. The University of North Carolina at Greensboro.

[51] Paniello-Castillo B, González-Rojo E, González-Capella T, Civit NR, Bernal-Triviño A, Legido-Quigley H, Gea-Sánchez M (2023). "Enough is enough": tackling sexism, sexual harassment, and power abuse in Spain's academia and healthcare sector. Lancet Reg Health Eur.

[52] Kennedy-Macfoy M (2024). On being fully human. Eur J Women's Stud.

